# Interactions between serotypes of dengue highlight epidemiological impact of cross-immunity

**DOI:** 10.1098/rsif.2013.0414

**Published:** 2013-09-06

**Authors:** Nicholas G. Reich, Sourya Shrestha, Aaron A. King, Pejman Rohani, Justin Lessler, Siripen Kalayanarooj, In-Kyu Yoon, Robert V. Gibbons, Donald S. Burke, Derek A. T. Cummings

**Affiliations:** 1 Division of Biostatistics and Epidemiology, University of Massachusetts, Amherst, MA 01002, USA; 2 Department of Epidemiology, Johns Hopkins University, Bloomberg School of Public Health, Baltimore, MD 21205, USA; 3 Department of Ecology and Evolutionary Biology, University of Michigan, Ann Arbor, MI 48109, USA; 4 Center for the Study of Complex Systems, University of Michigan, Ann Arbor, MI 48109, USA; 5 Department of Mathematics, University of Michigan, Ann Arbor, MI 48109, USA; 6 Fogarty International Center, National Institutes of Health, Bethesda, MD 20892, USA; 7 Queen Sirikit National Institute of Child Health, Bangkok, Thailand; 8 Department of Virology, Armed Forces Research Institute of Medical Sciences, Bangkok, Thailand; 9 University of Pittsburgh Graduate School of Public Health, Pittsburgh, PA 15261, USA

**Keywords:** dengue, infectious disease modelling, cross-protection, time-series models

## Abstract

Dengue, a mosquito-borne virus of humans, infects over 50 million people annually. Infection with any of the four dengue serotypes induces protective immunity to that serotype, but does not confer long-term protection against infection by other serotypes. The immunological interactions between serotypes are of central importance in understanding epidemiological dynamics and anticipating the impact of dengue vaccines. We analysed a 38-year time series with 12 197 serotyped dengue infections from a hospital in Bangkok, Thailand. Using novel mechanistic models to represent different hypothesized immune interactions between serotypes, we found strong evidence that infection with dengue provides substantial short-term cross-protection against other serotypes (approx. 1–3 years). This is the first quantitative evidence that short-term cross-protection exists since human experimental infection studies performed in the 1950s. These findings will impact strategies for designing dengue vaccine studies, future multi-strain modelling efforts, and our understanding of evolutionary pressures in multi-strain disease systems.

## 1. Introduction

For many multi-strain pathogens—e.g. influenza and RSV—research has shown evidence of immune response after infection with one strain that confers at least partial cross-protection against other strains of that pathogen [1,2]. For many multi-strain pathogens there is evidence of a broad, short-lived immune response immediately after infection with one strain that confers at least partial cross-protection against all strains of that pathogen. This short-term cross-protection plays a critical and central role in shaping the evolutionary and epidemiological dynamics of multi-strain pathogens. However, cross-protection introduces significant challenges to researchers looking to create an accurate and identifiable epidemiological model of disease. Short-term cross-protection can give rise to complex temporal dynamics in disease incidence, creating oscillating time series from which it is difficult to elicit information on or draw conclusions about parameters that govern the underlying epidemiological processes.

Dengue provides a particularly important and useful case study for a multi-pathogen disease system. Recently reported results from a dengue vaccine trial in Thailand highlight the importance of understanding the population-level landscape of susceptibility when evaluating the efficacy of a vaccine with enormous potential to reshape the global dynamics of dengue [3]. In a prime example of how cross-protection can create difficulties in epidemiological analysis, this trial faces substantial inferential challenges that may make it difficult to draw conclusions about the efficacy of a vaccine when naturally acquired cross-protection serves as a pre-existing immunization of a large portion of the population. Having an estimate of the duration of cross-protection can assist in planning studies to adequately control for this existing immunity.

Cross-reactive and non-specific immune responses are thought to be principal drivers of diversity for multi-strain pathogens [4,5]. Among human pathogens, dengue is unusual in how it exists in a small number of antigenic groups (the four serotypes) for a long period of time. Many other pathogens have either a large (greater than 50) and diverse population of antigenically distinct strains coexisting for long periods of time (as with pneumococcus [6], meningococcus [7], malaria [8] and rhinoviruses [9]), or a very small number of antigenically distinct strains where new groups rapidly displace old ones (as with influenza). Therefore, in the context of modelling interactions between strains, dengue occupies a sweet-spot of genetic diversity (not too many co-circulating serotypes, but not too few) that provides us with fertile ground for developing a model that can estimate these interactions.

The four antigenically distinct serotypes of dengue virus (DEN-1,…, DEN-4) have co-circulated in Bangkok for over 60 years. These viruses cause substantial morbidity and mortality globally [10]. Although there is genetic variation within serotypes, they are more genetically stable than many other RNA viruses, with an estimated 1 nucleotide change per year [11]. Exposure to one virus induces lifelong immunity to all other members of that virus's serotype [12], but the interaction between serotypes is less clear. The co-circulation of these genetically stable viruses allows us to estimate their interaction over long timescales and investigate the impact of specific human immune responses on the competition between viruses.

Secondary infections with dengue virus are more severe than primary infections. Multiple hypotheses have been proposed to explain the mechanism of this feature of dengue pathogenesis. A common explanation focuses on antibody-dependent enhancement [13,14]. Researchers have proposed that antibody concentrations are nonlinearly associated with protective immune responses [15]. Specifically, this recent work has hypothesized that high concentrations are protective, low concentrations are irrelevant and mid concentrations place people at risk through antibody-dependent enhancement. Since antibody levels wane over time, the timing of the last exposure can dictate whether a new exposure results in heterologous immunity or more severe infection [16,17]. The epidemiological impact of the immunological interaction between dengue serotypes has been the focus of a large number of manuscripts [18–24]. Many prospective cohort and simulation studies have focused on the enhancement of disease and/or transmissibility upon secondary infection [18,19,25]. Models of dengue transmission have suggested that inclusion of short-term cross-protection is necessary to reproduce the temporal dynamics of dengue incidence based on qualitative aspects of the dynamics of incidence [21–23]. However, none of these studies makes more than qualitative comparison of models with and without cross-protection and none provides a quantitative estimate of the strength and duration of cross-protection.

Albert Sabin conducted the first studies to show short-term cross-protection of humans, who had been infected with one dengue virus to infection with a different (or heterologous) dengue virus. Humans infected with DENV-1 or DENV-2 were protected from clinical illness when challenged (by subcutaneous injection) with heterologous virus within two months of primary experimental infections [26,27]. Two to three months after primary experimental exposure, some subjects developed viraemia upon exposure to a heterologous virus. For up to nine months, some subjects experienced either no illness or a more mild illness upon secondary exposure compared with primary exposure. Sabin's statements in his paper and in papers obtained from his archives do not make it clear whether this duration was the longest interval observed or the longest interval tested among his subjects [26,27]. Cross-protection has also been observed in experimental infections of primates [28]. A few other observational studies have found evidence of cross-protection between serotypes [24,29]. Case fatality rates were significantly reduced among individuals experiencing a secondary infection with DENV-2 4 years after a primary DENV-1 infection compared with those experiencing a secondary DENV-2 infection 20 years after a primary DENV-1 infection [30]. Kliks *et al.* [29] found that children who experienced a mild illness upon dengue infection had greater levels of heterotypic neutralizing antibodies than those who experienced a hospitalized illness upon infection. However, no longitudinal studies have been conducted to characterize short-term cross-protection between each of the dengue serotypes.

We compare the ability of multiple novel mechanistic transmission models to predict dengue incidence using past incidence in a 38-year time series of serotype-specific incidence. We use these models to estimate the duration and strength of short-term cross-protection between dengue serotypes using serotype-specific incidence data from a reference hospital for dengue in Bangkok, Thailand. We show that models that include short-term cross-protection perform significantly better than those that do not and present quantitative estimates of the duration of short-term cross-protective immunity.

## 2. Material and methods

### 2.1. Four decades of monthly case-count data from Bangkok

**Figure 1.**
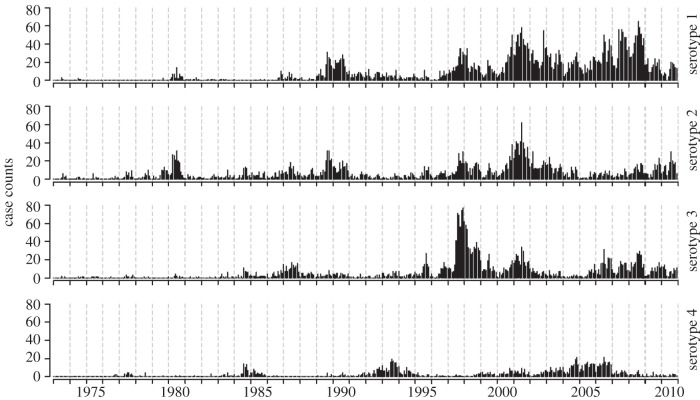
The time series of monthly serotype-specific case counts of dengue from Queen Sirikit National Institute of Child Health in Bangkok, Thailand. This facility is a paediatric healthcare facility which serves as a reference hospital for dengue in Bangkok. The counts shown here represent the total number of cases, both primary and secondary infections.

Our dataset consists of monthly case counts of serotype-specific, laboratory confirmed dengue illness for 38 years, from 1973 through 2010, reported by the Queen Sirikit National Institute of Children's Health in Bangkok, Thailand. This facility is a paediatric healthcare facility, which serves as a reference hospital for dengue in Bangkok. Approximately 10% of hospital-attended dengue disease in Bangkok is cared for at this hospital. The data were reported as monthly case counts of both primary or secondary infections. Laboratory methods changed several times over the course of data collection, most dramatically in 1981 and 1995 [31]. Figure 1 shows the time series of monthly case counts, by serotype. Each serotype-specific monthly time series was interpolated into biweeks using restricted cubic splines fit to the series of cumulative counts. We also compiled data on yearly births in Bangkok for 1973 through 2010. Data were not available for 1973 so we interpolated the yearly birth count for this year. Biweekly births were interpolated from the annual data. During the period studied infant mortality was low, indicating that any impact on our modelling of the susceptible population would be minimal [32].

### 2.2. Adapted time-series susceptible-infected recovered model

We have adapted the time-series susceptible-infected recovered (TSIR) model, developed by Finkenstädt & Grenfell [33]. We assume a transmission model of2.1
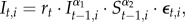
where *I_t,i_* is the count of infecteds at time *t* for serotype *i*, *S_t,i_* is the count of susceptibles and *r_t_* is a transmission parameter that is time-varying with period of 1 year. The *α* are mixing parameters, which, if both equal 1, define a population with homogeneous mixing, whereas values not equal to 1 have been used to describe departures from mass-action mixing [34] and to account for discretization of continuous-time transmission processes [35]. It is assumed that the error term *ε_t,i_* has the following properties: 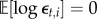 and 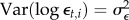. We used a time-step of two weeks (a ‘bi-week’) which roughly coincides with the generation time of dengue fever. Additionally, we accounted for multi-strain susceptible dynamics according to the following equation:2.2
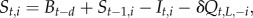
where *B_t–d_* is the number of births entering the susceptible cohort at time *t*, after receiving an assumed *d* = 8 bi-weeks of maternal antibody protection. Also, *Q_t,L,–i_* is a term which accounts for the transitions between susceptible and convalescent states for a particular strain *i*. This term depends on the parameters, the specified model of cross-protection (*k* or *λ*, for fixed duration and exponential models, respectively) as well as the maximum possible length of cross-protection (*k* or *L*). More details about the susceptible accounting can be found in the electronic supplementary material, §1. The parameter *δ* is the fraction of infected individuals that gain transient immunity to all other serotypes—i.e. are removed from the susceptible population for other serotypes—for some period of time.

The model represents the possible trajectories of individuals through particular states regarding their infection with each serotype (presented in electronic supplementary material, figure S6). Individuals begin life susceptible to all serotypes and become infected with each serotype over the course of their lifetime. Mosquitos are represented implicitly in our model. The potentially time-varying transmission parameter, *r_t_*, describes the rate at which the multiple processes involved in transmission (i.e. mosquito feeding, viral growth in mosquitos, mosquito survival) gives rise to new infections in humans. Upon infection, individuals become temporarily immune to infection by the other serotypes. That is, those infected with strain *j* are removed for a period of time from the susceptible class for strain *i*, where *j* ≠ *i*, to a convalescent, cross-protected class CP. This state could represent protection from infection or protection from symptomatic, hospital-attended clinical disease. Importantly, we assume that people can be infected after leaving this temporarily cross-protected state. Thus, if only cross-protected from clinical disease, we assume that a subclinical illness does not elicit a protective immune response to the infecting serotype. We are interested in estimating parameters that govern the length and duration of this state of cross-protection.

### 2.3. Parametric forms of cross-protection

We assumed two parametric forms for the period of cross-protection. We refer to the first parametrization as a ‘fixed duration’ model. In these models, we assume that some fraction *δ* of all infected individuals experience a period of cross-protection with a fixed length, *k*. Fixed duration models experienced some problems with convergence for some of the large values of *k* included in the analysis. For this reason, the range of *k* was truncated to be 100 biweeks, which included the 90% and 95% confidence regions for *k* and *δ*. We refer to the second parametrization as ‘exponential’ models. In these models, we assume that infected individuals leave the cross-protected class according to a random exponential survival distribution with mean *λ*. The exponential model could also be described as having protection in individuals wane over time. We truncate this distribution, assuming that the maximum amount of time a person can be cross-protected is equal to *L*, which was fixed at 10 years and five months. This length was decided upon because it was the 75th percentile of the exponential distribution with a mean of 7.5 years—the highest *λ* considered. It is necessary to truncate the distribution because this determines how much data are needed to ‘seed’ the initial time-step of the model.

As a sensitivity analysis, we allowed *δ* to vary between 0 and 1 in our exponential models. The fitted models yielded a maximum-likelihood estimate of *δ* equal to 1, indicating that our original single-parameter model incorporates parsimoniously exponentially distributed durations of cross-protection.

### 2.4. Statistical methods and sensitivity analyses

We adapted the TSIR methodology to account for the potential impact of cross-protection. The optimal parameters were identified by maximizing a likelihood function which we calculated for each point on a grid of plausible parameter values. The likelihood function required an iterative algorithm to estimate the case reporting fractions which was followed by fitting a log-linear regression model. Confidence regions for the resulting likelihood surface were determined using appropriate *χ*^2^-tests [36]. To evaluate the relative goodness of fit between the fitted models, we used likelihood ratio tests and Akaike Information Criterion (AIC) [37]. Bayesian Information Criterion (BIC) was also considered, but it appeared to penalize the models too strongly for additional parameters (see the electronic supplementary material, table S1) and has not been recommended for ecological modelling contexts [38]. We also computed the Pearson correlation coefficients between the observed data and rescaled predictions (based on the estimated reporting fraction). A detailed description of the methodology is available in the electronic supplementary material, §1.

We tested our methods on simulated data from a system developed independently by a subset of co-authors [39]. We generated simulated datasets by varying three parameters: the reporting fraction (either 10 or 1%), the average duration of cross-protection (1 day, 6 months, 1 year, 1.5 years, 2 years or 3 years) and the inclusion of susceptible enhancement (included as either a 40% increased risk of secondary infection or no increased risk). For each of the 24 possible combinations of parameters, 500 datasets were simulated for a total of 12 000 datasets. Neither seasonal nor serotype-specific variability in transmission rates were included in the data-generating model. Detailed simulation study methodology is available in the electronic supplementary material, §1.

We repeatedly simulated serotype-specific incidence over the time-period of the observed data. To compare the impact that cross-protection has on the multi-annual dynamics of the overall system, we simulated 1000 datasets from our best-fitting fixed duration cross-protection model, *F_c_*, and 1000 datasets from our best-fitting model that did not include cross-protection, *N_c_*. The data were simulated on the scale of the fully observed and unreported data and subsequently down-sampled to be on the scale of the observed case counts. For each simulated dataset, we computed the Fourier spectrum. Before calculating the spectra, the simulated data were detrended and normalized. More methodological details about this simulation study are available in the electronic supplementary material, §1.

All analyses were performed in R, v. 15.1 [40]. Datasets and code needed to reproduce the results presented here are available on github at https://github.com/nickreich/dengueCP.

## 3. Results

Serotype-specific case counts from Queen Sirikit National Institute of Children's Health in Bangkok, Thailand are shown in figure 1. Cases of each serotype show periodic behaviour as measured by Fourier spectra at periods of 8–12 years (see the electronic supplementary material, figures S1 and S2). Total incidence shows periodic oscillations of 3–5 years (see the electronic supplementary material, figures S1 and S2).

### 3.1. Existence of cross-protection is supported by data

**Figure 2.**
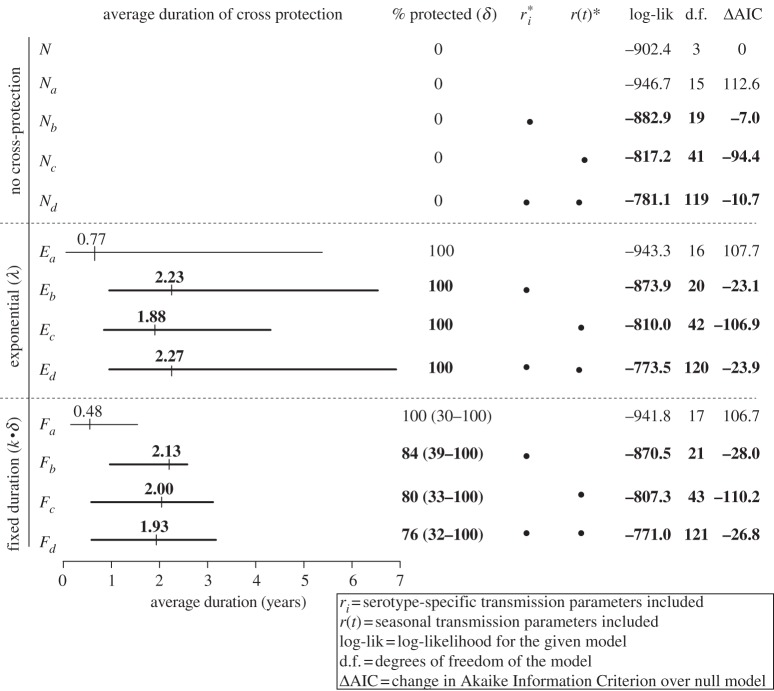
Estimated parameters and model characteristics for exponential and fixed duration models of cross-protection. Model results shown in bold indicate that the model showed a statistically significant improvement over a null model that included no form of cross-protection and no serotype-specific or seasonal transmission parameters. Based on AIC and likelihood ratio tests, models *E_c_* and *F_c_* showed the most improvement over the null model and also showed significantly better fit to the data than a model that did not include cross-protection but did include seasonal transmission. The point estimates of the average duration of cross-protection are shown by the vertical tick mark and the 95% CIs are shown with horizontal lines.

Models that included short-term cross-protection either as having a fixed or an exponentially distributed duration fit our data better than models without cross-protection, based on likelihood ratio tests comparing our fitted models. Our results for all fitted models are summarized in figure 2 and presented in full in electronic supplementary material, table S1. Models were assessed by their ability to predict incidence in each bi-week of the time series. We chose bi-weeks as the time-step of the model because two weeks corresponds roughly to the estimated generation time—the time between successive infections in a chain of transmission—for dengue.

Assuming a fixed duration of protection follows some fraction of primary infections, the best-fitting model (referred to as *F_c_* in figure 2 and electronic supplementary material, table S1) estimated that 80 per cent (95% CI 33–100) of infected individuals are protected for a duration of 2.5 years (95% CI 1.35–3.31). This corresponds to an average duration of cross-protection (averaged across those with and without cross-protection) of 2.00 years (95% CI 0.75–3.10). Assuming that all individuals experience an exponentially distributed duration of cross-protection, the best-fitting model (*E_c_*) estimated an average duration of cross-protection to be 1.88 years (95% CI 0.88–4.31). Figure 2 shows the average duration of cross-protection for fixed duration models (averaged across those with and without protection) for comparison with exponential distributed durations. These models show high correlation with observed data (see the electronic supplementary material, table S1).

**Figure 3.**
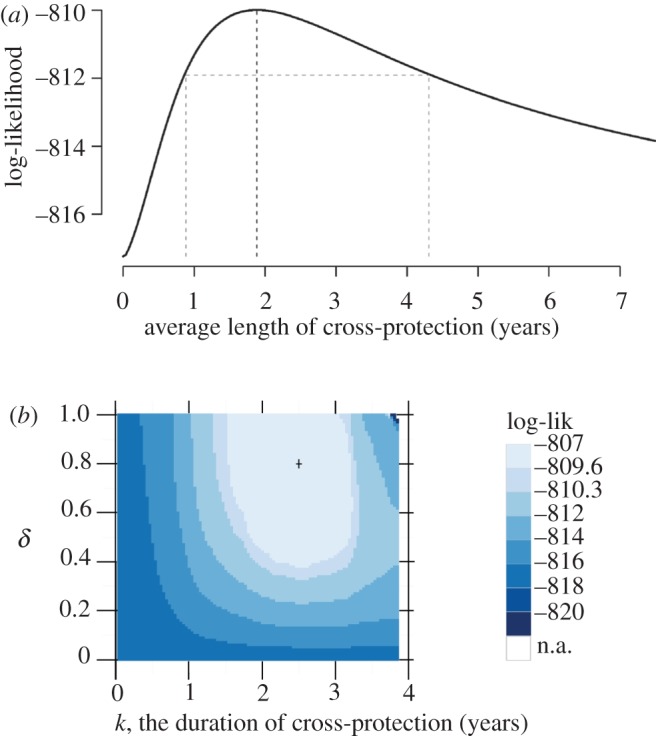
Profile likelihood surfaces for the best exponential models (*a*) and fixed duration models (*b*). In panel (*a*), the maximum-likelihood and 95% CI for *λ* are shown by the dashed vertical lines. In (*b*), the axes index the two parameters of a fixed duration distribution of cross-protection. The fraction of the population that experiences cross-protection is *δ* and *k* is the duration of cross-protection. The two lightest regions represent the 90% and 95% likelihood confidence regions. The confidence region for the average duration (which is calculated as *k · δ*) represents the range of average durations contained in the respective confidence region. (Online version in colour.)

The profile likelihood of the observed data as a function of parameters describing cross-protection is presented for the two best models in figure 3. These two models fit the data better than all models that did not include any form of cross-protection (models *N*, *N_a_*, *N_b_*, *N_c_* and *N_d_*), including ones with seasonal transmission (*N_c_* and *N_d_*). Figure 2 displays for all exponential and fixed duration models the log-likelihood of the full model, and the change in AIC when compared with the simplest model, *N*. By AIC, models *F_c_* and *E_c_* fit the data best. Additionally, the likelihood ratio test statistics of model *F_c_* to model *N_c_* is 20.2 with 2 d.f. and of model *E_c_* to model *N_c_* is 14.5 with 1 d.f., indicating a significant increase in model performance with the inclusion of either form of cross-protection (*p*-values from each *χ*^2^-likelihood ratio test less than 0.001).

### 3.2. Factors impacting disease transmission

The inclusion of serotype-specific transmission coefficients improved the fit of models compared with the null model (AIC for model *N* = 1811, while AICs for models *N_b_*, *F_b_* and *E_b_* were 1803, 1783 and 1788, respectively). Including both serotype-specific and seasonal transmission rates also showed an improvement over the null model (AICs for models *N_d_*, *F_d_* and *E_d_* were 1800, 1784 and 1787, respectively). However, according to AIC and likelihood ratio tests, the most parsimonious models were *N_c_*, *F_c_* and *E_c_* which included only seasonal transmission rates (AICs for models *N_c_*, *F_c_* and *E_c_* were 1716, 1701 and 1704, respectively).

**Figure 4.**
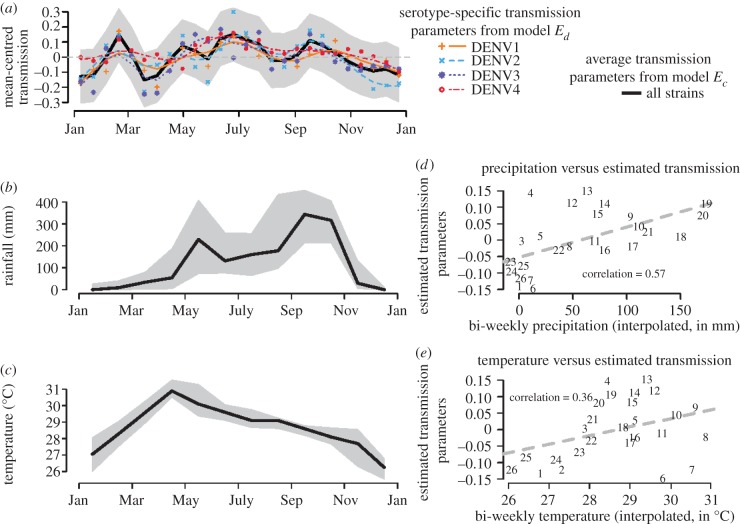
Estimated bi-weekly transmission parameters from the best-fitting exponential model of cross-protection. Temperature and rainfall data provide a comparison with the observed seasonal trends. (*a*) Displays mean-centred estimated bi-weekly transmission parameters. The transmission across all serotypes (from model *E_c_*) is shown in the solid black line with the grey shaded area representing the 95% CI. Serotype-specific transmission parameters (from model *E_d_*) are shown in the thinner coloured lines. Transmission parameters from the corresponding fixed duration models showed similar patterns. (*b*) Median monthly precipitation in millimetre, at one location in Bangkok between 1981 and 2002. (*c*) Median monthly temperature in Bangkok between 1983 and 1996. The shaded areas in (*b*,*c*) Represent the 10th and 90th percentiles of the observed monthly data. (*d*) Plots the bi-weekly transmission parameters against the average bi-weekly precipitation and a ‘line of best fit’ is shown. (*e*) Plots the bi-weekly transmission parameters against the average bi-weekly temperature and a ‘line of best fit’ is shown. For (*d*,*e*), Cubic splines were used to interpolate the monthly data in (*b*,*c*) into bi-weekly data. In (*d*,*e*), the points are the graph are indicated by the corresponding bi-week (numbered 1–26). (Online version in colour.)

The inclusion of serotype-specific and/or seasonal transmission coefficients resulted in longer estimates of the duration of cross-protection. Figure 4 shows the pattern of time-varying or seasonal transmission coefficients fit for each serotype and aggregated across all serotypes for the exponential cross-protection models. Seasonal transmission coefficients varied from 84% of the mean value at its peak to 116% of its mean value at its trough. There is general correspondence between peaks in temperature, rainfall and the high values of the transmission coefficient, but several peaks occur during dry and cool periods as well (figure 4).

### 3.3. Model generates 3–5 year multi-annual patterns

To investigate whether our estimated models could generate multi-annual patterns displayed in the observed incidence data, we simulated serotype-specific incidence over the time-period of the observed data. Using Fourier transforms, we estimated the frequency of multi-annual oscillations in the simulated data and compared this with empirical data. Details on these simulations are included in the electronic supplementary material, §1. Simulated datasets from systems with a fixed duration of cross-protection showed strong evidence of annual cycles as well as 3–5 year serotype-specific multi-annual cycles. These frequencies roughly align with periodicities observed in the actual data, although they fail to capture some of the observed longer term dynamics. In contrast, not including cross-protection resulted in no consistent multi-annual oscillations. Electronic supplementary material, figure S2 shows the serotype-specific spectra for the observed data as well as for the simulated datasets.

### 3.4. Multi-strain model framework is sensitive to cross-protection

To assess our estimation procedure, we fit our models to data generated from simulations in which the true parameters were known. Using the multi-strain TSIR framework, we analysed 12 000 simulated datasets from a continuous-time multi-strain state-space model that incorporates exponentially distributed cross-protection and susceptibility enhancement (i.e. individuals with immunity to one serotype are more likely to acquire a second) [39]. For each simulated dataset, the 13 candidate models were fitted and the best-fitting model was chosen as the one with the lowest AIC. The TSIR model was both sensitive and specific in identifying the presence or the absence of cross-protection in simulated datasets, based on the results from the best-fitting models. The TSIR model had 77 per cent specificity—correctly identifying that no cross-protection was present in 77 per cent of the datasets that had none. The TSIR model also had 100 per cent sensitivity—identifying a significant level of cross-protection in all datasets that came from a system with some level of cross-protection. We analysed simulated data with varying reporting rates (10 and 1% of all cases reported) to test the robustness of our estimates to only observing a fraction of all cases. In 869 of 1000 datasets with no cross-protection where 10 per cent of all cases were assumed to be reported, the TSIR model correctly identified that no cross-protection was present. When 1 per cent of cases were assumed reported, the TSIR model correctly identified no cross-protection in 667 of 1000 simulated datasets. Detailed methodology and results from the simulation are presented in table S3, electronic supplementary material, figures S3 and S4 and §2.

### 3.5. Consideration of immune enhancement

Models *F_a_*, *F_b_*, *F_c_* and *F_d_* restricted the parameter *δ* to lie between 0 and 1. In this case, *δ* can be interpreted as the fraction of infected individuals who experience some level of cross-protection. The models we present in this manuscript focus on these cross-protection interpretations of *δ*. However, allowing *δ* to assume negative values admits another interpretation, namely that *δ* describes the relative contribution of those individuals infected with one serotype to a population's susceptibility to a different serotype. In this scenario, negative values of *δ* are consistent with immune enhancement of severity of or susceptibility to infections among those previously infected. In a simple sensitivity analysis that extended our primary models, we explored the range of *δ* values from 0 to −1 in model *F_c_*. These results are presented in electronic supplementary material, figure S5. For periods of 0–4 years, we did not find any support for increased contribution of recently infected individuals to the pool of susceptible individuals.

## 4. Discussion

It has been proposed that the phylogenetic structure of dengue viruses reflects competing processes. On the one hand, cross-protective immunity may increase the genetic distance between viruses within different serotypes by selecting for genetic variants that escape cross-protective immunity [22]. On the other hand, antibody-dependent enhancement may only occur in viruses that are antigenically similar [41]. Here, we have provided strong evidence that at least one of these processes (cross-protection) plays an important role in the transmission dynamics of each dengue serotype.

We find strong evidence for short-term cross-protection of a duration of approximately 2 years. Our results were robust to different assumptions of the distribution of cross-protection, seasonality of transmission and heterogeneity in transmission between serotypes. These estimates provide the first quantitative estimate of the duration of short-term cross-protection between dengue serotypes since Albert Sabin's experimental data collected in the 1940s.

The data that we have analysed do not allow us to distinguish between cross-protection against infection and cross-protection against clinically apparent disease. Moreover, the medically attended dengue incidence considered here may be strongly correlated with the predominant serotypes circulating in the population or it may have a more complicated relationship with serotype-specific incidence of infection. Heterogeneity in severity by serotype or infection sequence (e.g. secondary infections of type *j* following type *i*) may threaten our ability to estimate durations of cross-protection and other transmission parameters, because it reduces the correlation between observed clinical case data and the temporal incidence of infection. Longitudinal observations of the incidence of infection are needed in order to determine the causal mechanisms underlying our observations. Though longitudinal cohort data would be ideal for resolving specific mechanisms of interaction, the expense of collecting this data means that long durations (on the order of 40 years) have not and will not be performed. Our data have allowed us to model and estimate non-specific serotype interactions over four decades.

Given that secondary infections with dengue are more likely to result in severe illness than primary infections, any hospital-based case data (such as the data used in this analysis) will have an over-representation of secondary infections. Temporal differences in primary and secondary infection dynamics could impact our ability to accurately estimate the duration of cross-protection. This is a limitation of the work presented here. However, research that has shown that primary dynamics are tightly coupled to secondary dynamics across a wide range of theoretical dengue models [19,20] suggests that bias introduced by unrepresentative primary and secondary case sampling may be minimal.

We assume that individuals can be infected with only one serotype at a time. Co-infections of multiple dengue serotypes have been observed to occur [42,43], though one study has suggested that competitive interactions between the serotypes within mosquitoes may limit the transmission of multiple types [44]. We would expect the inclusion of this possibility would increase our estimates of the duration of short-term cross-protection.

Several recent studies have found evidence to support the existence of cross-protection between dengue serotypes. The durations that these papers suggest are 5–12 months [45], 1–3 years [24] and four months to 9 years [12]. Other research has provided evidence that the presence of heterologous neutralizing antibody titres and the pathogenicity of secondary infections are correlated with the timing of a primary infection [46,47]. However, none of these papers explicitly estimate a duration of cross-protection. Instead, they estimate the length of time between subsequent infections in individuals [12,24], or the length of time separating the occurrence of different serotypes at a population level within a small spatial scale [45]. Our estimates of the duration of cross-protection are considerably longer than Dr Sabin's estimate of ‘two to nine months’; however, these results are difficult to compare as they exposed individuals by a route different from natural exposure and little detail is available on the experiments that led to this observation [27].

A strength of the work presented here is that we have created a new, robust framework that allows us to derive data-driven estimates of the duration of cross-protection between dengue serotypes. Few dynamical models of this complexity have been fit to serotype-specific dengue time-series data, and none to our knowledge has explicitly estimated the duration of cross-protection. We anticipate that this will be an area of active research in coming years, as researchers bring more data to bear on the problem and methods are improved. While our results are robust to a wide range of assumptions about how the reporting rates change over the study period (see the electronic supplementary material for a more complete discussion), this is a particular area of this framework that we feel could benefit from future research. For example, pairing observed, non-serotype-specific case data with serotype-specific case counts could, if the additional uncertainty is managed appropriately, allow for larger number of cases to be used for these models.

Including seasonal transmission coefficients in our models improved the fit to data compared with models without this feature or with serotype-specific (but time-constant) transmission coefficients. Interestingly, our estimated pattern of seasonal transmission coefficients does not show one clear peak, but several over the course of the year (figure 4). With the exception of two bi-weeks in February and March, estimated transmission parameters are below-average between October and May; however, the confidence intervals for the mean-centred transmission parameters all include zero. A model that analysed serotype-specific incidence data from multiple locations could explicitly estimate the degree to which environmental factors such as rainfall and temperature impact transmission of dengue.

Providing additional validation of the importance of short-term cross-protection, the inclusion of cross-protection leads to simulations that qualitatively match some of the observed multi-annual patterns in incidence data better than models that do not include cross-protection. However, some model mis-specification is present, revealed by the fact that the multi-annual patterns seen in simulated data align well with the shorter term oscillations seen (on the order of 3–5 years) but they miss longer term dynamics on the scale of 8–15 years. Thus, there is room for improvement. A question for future research is the extent to which these observed multi-annual signatures are governed by the choice of parameters that define the duration and strength of cross-protection.

We considered a discrete-time model which simplifies aspects of the transmission process that may be very important. Our simulation studies are encouraging in that we were able to successfully estimate the duration of cross-protection in data generated by models that differed in structure considerably from our assumed model. The models used to create simulated datasets were continuous-time models that incorporated immune enhancement of susceptibility to infection. By adapting existing methods for analysing infectious disease time-series data, we have created and applied a novel framework to estimate the duration and strength of cross-protection between dengue strains. This methodology could be extended to other disease settings or adapted to incorporate further hypothesized interactions (e.g. immune enhancement) between pathogenic strains.

Our results provide support to efforts to estimate the potential population-level effects of dengue vaccination. The duration and strength of cross-protection are fundamental drivers of incidence data and could provide different estimates of the impact of wide-scale immunization at different settings. Additionally, our results suggest that immunization trials may need to be continued for multiple years in order to understand the long-term impact of immunization as naturally acquired short-term immunity in a vaccinated population wanes.
